# Supplementary material for: A hierarchical Bayesian latent class mixture model with censorship for detection of linear temporal changes in antibiotic resistance
Source: PLoS One. 2020 Jan 31;15(1):e0220427. doi: 10.1371/journal.pone.0220427 (PMC6993983; doi:10.1371/journal.pone.0220427)
Supplement: S1 Appendix — (PDF) [file pone.0220427.s001.pdf]

S1 Appendix. Gibbs sampling procedure. The Gibbs sampling procedure was conducted as described here. We used "·|·" to denote full conditional distribution unless otherwise specified. This parameter is the distribution of what is before the pipe and is conditional on all other parameters involved in the model. In the following steps, j = 1, . . ., n_i, and i = 1, . . ., I.

1. Obtain draws of latent continuous variable y_{ij} from censored observation y^*_{ij} using the inverse cumulative distribution function (inverse CDF) method. By observing y^*_{ij}, we sampled from the full conditional normal distribution with the boundaries l_{ij} and u_{ij}. To be more specific, the three censoring situations are discussed below:

   - When y^*_{ij} is interval censored with limits l_{ij} and u_{ij}, y_{ij} is updated via
   $$y_{ij} = \Phi^{-1}\{\Phi(l_{ij}) + U[\Phi(u_{ij}) - \Phi(l_{ij})]\}, \tag{15}$$
   where Φ is the CDF function of standard normal distribution, and Φ^{-1} is the inverse of the CDF function. U is a random draw from $Unif(0, 1)$.

   - When y^*_{ij} is left censored with limits $l_{ij} = -\infty$ and u_{ij}, y_{ij} is updated via
   $$y_{ij} = \Phi^{-1}\{U[\Phi(u_{ij})]\}. \tag{16}$$

   - When y^*_{ij} is right censored with limits l_{ij} and $u_{ij} = \infty$, y_{ij} is updated via
   $$y_{ij} = \Phi^{-1}\{\Phi(l_{ij}) + U[1 - \Phi(l_{ij})]\}. \tag{17}$$

2. Draw samples of c_{ij} from their full conditional distribution
$$c_{ij}| \cdot \overset{ind}{\sim} Ber(h_{ij}), \tag{18}$$
where $h_{ij} = \frac{p_i \Phi_1(y_{ij}|\beta_{1i},\sigma_1^2)}{(1-p_i)\Phi_0(y_{ij}|\beta_{0i},\sigma_0^2)+p_i\Phi_1(y_{ij}|\beta_{1i},\sigma_1^2)}$, describing the chance for an observation to be from the resistant population. $\phi(y|\beta, \sigma^2)$ represents the probability density function of a normal distribution with mean β and variance σ^2.

3. Sample the intercept parameter γ_0 in the linear part from its full conditional distribution
$$\gamma_0| \cdot \sim N(m_0, v_0), \tag{19}$$
where $v_0^{-1} = \frac{I}{\tau_0^2} + \frac{1}{c'}$, $m_0 = v_0 \cdot \left[\frac{\sum_{i=1}^{I} \beta_{0i} - \gamma_1 t_i}{\tau_0^2} + \frac{\mu'}{c'}\right]$, $\mu' = 0$ is the prior mean of γ_0, and $c' = 10^6$ is the prior variance of γ_0.

4. Sample the slope parameter γ_1 in the linear part from its full conditional distribution
$$\gamma_1| \cdot \sim N(m_i, v_i), \tag{20}$$
where $v_i^{-1} = \frac{t_i^2}{\tau_0^2} + \frac{1}{c'}$, $m_i = v_i \cdot \left[\frac{\beta_{0i} - \gamma_0}{\tau_0^2 t_i} + \frac{\mu'}{c'}\right]$, $\mu' = 0$ is the prior mean of γ_1, and $c' = 10^6$ is the prior variance of γ_1.
Starting from $i = 1$, for $i < I$, return to step 3 and 4 to sample linear parameters for another time. Increase i by 1. When i reaches I, continue to step 5.

5. Sample μ_1, the hierarchical yearly mean of the mean $\log_2$MIC in the resistant population, from its full conditional distribution

$$\mu_1 | \cdot \sim N(m_{\mu_1}, v_{\mu_1}), \tag{21}$$

where $v_{\mu_1}^{-1} = \frac{I}{\tau_1^2} + \frac{1}{c'}$, $m_{\mu_1} = v_{\mu_1} \cdot \left[\frac{\sum_{i=1}^{I} \beta_{1i}}{\tau_1^2} + \frac{\mu'}{c'} \right]$, $\mu' = 0$ is the prior mean of μ_1, and $c' = 10^6$ is the prior variance of μ_1.

6. Sample the real yearly mean $\log_2$MIC in the non-resistant population, β_{0i}, from its full conditional distribution

$$\beta_{0i} | \cdot \sim N(M_0, V_0), \tag{22}$$

where $V_0^{-1} = \frac{\sum_{j=1}^{n_i} I(c_{ij}=0)}{\sigma_0^2} + \frac{1}{\tau_0^2}$ and $M_0 = V_0 \cdot \left[\frac{\sum_{j=1}^{n_i} y_{ij} I(c_{ij}=0)}{\sigma_0^2} + \frac{\mu_{0i}}{\tau_0^2} \right]$.

7. Sample the real yearly mean $\log_2$MIC in the resistant population, β_{1i}, from its full conditional distribution

$$\beta_{1i} | \cdot \sim N(M_1, V_1), \tag{23}$$

where $V_1^{-1} = \frac{\sum_{j=1}^{n_i} I(c_{ij}=1)}{\sigma_1^2} + \frac{1}{\tau_1^2}$ and $M_1 = V_1 \cdot \left[\frac{\sum_{j=1}^{n_i} y_{ij} I(c_{ij}=1)}{\sigma_1^2} + \frac{\mu_1}{\tau_1^2} \right]$.

8. Sample σ_l^2, variance of latent $\log_2$MIC, in either population from its full conditional distribution

$$\sigma_l^2 | \cdot \sim IG\left(a + \frac{1}{2} \cdot \sum_{i=1}^{I} \sum_{j=1}^{n_i} I(c_{ij=l}), b + \frac{1}{2} \sum_{i=1}^{I} \sum_{j=1}^{n_i} I(c_{ij} = l)(y_{ij} - \beta_{li})^2 \right), \tag{24}$$

where $l = 0$ represents the non-resistant population, and $l = 1$ represents the resistant population. $a = 10^{-4}$ is the prior shape parameter, and $b = 10^{-4}$ is the prior rate parameter.

9. Sample τ_0^2, the variance for the mean $\log_2$MIC in the non-resistant population, from its full conditional distribution

$$\tau_0^2 | \cdot \sim IG\left(a + \frac{I}{2}, b + \frac{\sum_{i=1}^{I} (\beta_{0i} - \gamma_0 - \gamma_1 t_i)^2}{2} \right), \tag{25}$$

where $a = 10^{-4}$ is the prior shape parameter, and $b = 10^{-4}$ is the prior rate parameter.

10. Sample τ_1^2, the variance for the mean $\log_2$MIC in the resistant population, from its full conditional distribution

$$\tau_1^2 | \cdot \sim IG\left(a + \frac{I}{2}, b + \frac{\sum_{i=1}^{I} (\beta_{1i} - \mu_1)^2}{2} \right), \tag{26}$$

where $a = 10^{-4}$ is the prior shape parameter, and $b = 10^{-4}$ is the prior rate parameter.

11. Obtain draws of parameter θ from its full conditional distribution

$$\theta | \cdot \sim N(m_\theta, v_\theta), \tag{27}$$

where $v_\theta^{-1} = \frac{I}{v^2} + \frac{1}{c'}$, $m_\theta = v_\theta \left[\frac{\sum_{i=1}^{I} \alpha_i}{v^2} + \frac{\mu'}{c'} \right]$, $\mu' = 0$ is the prior mean of θ, and $c' = 10^6$ is the prior variance of θ.

12. Sample v^2, the variance of α_i, from its full conditional distribution

$$v^2 | \cdot \sim IG\left(a + \frac{I}{2}, b + \frac{\sum_{i=1}^{I} (\alpha_i - \theta)^2}{2}\right), \tag{28}$$

where $a = 10^{-4}$ is the prior shape parameter, and $b = 10^{-4}$ is the prior rate parameter.

13. Sample α_i, the log-odds of the proportion, using random walk Metropolis-Hastings from their posterior to some constant

$$f(\alpha | \mathbf{c_i}) \propto \frac{exp(\alpha_i \sum_{j=1}^{n_i} c_{ij})}{1 + exp(\alpha_i)} exp\left(-\frac{(\alpha_i - \theta)^2}{2v^2}\right), \tag{29}$$

where $\mathbf{c_i}$ is the vector of c_{ij}, $j = 1, \ldots, n_i$.
