# Supplementary material for: A hierarchical Bayesian latent class mixture model with censorship for detection of linear temporal changes in antibiotic resistance
Source: PLoS One. 2020 Jan 31;15(1):e0220427. doi: 10.1371/journal.pone.0220427 (PMC6993983; doi:10.1371/journal.pone.0220427)
Supplement: S1 File — The certificate of English editing is attached in the Supporting information as an external file. (PDF) [file pone.0220427.s002.pdf]

CERTIFICATE OF EDITING

To whom it may concern:

This letter confirms that the manuscript detailed below was edited by the professional English-language scientific editing staff at BioScience Writers, LLC. A copy of this manuscript as edited is available at our website. Enter the validation code provided below in the entry field and click "Verify" to view the final edited version of this document.

Best Regards,
Scientific Editing Staff
BioScience Writers, LLC

Thursday, June 27, 2019

Manuscript Title:

A Hierarchical Bayesian Latent Class Mixture Model with Censorship for Detection of Linear Temporal Changes in Antibiotic Resistance

Manuscript Author(s):

Annette O'Connor

Editing Service Level Selected by Client:

1. Basic

Validation Code:

9E3B6F76-DBCE-404A-B518-5EC8E3B1B9A6

To validate this certificate visit: www.biosciencewriters.com/verify.

Use of this certificate grants BioScience Writers, LLC permission to share the final version of manuscript files with journals upon request. BioScience Writers makes no claim as to the accuracy of the research content or objectives of the author. The text as edited is grammatically correct. The authors have the option to accept or reject suggestions and changes to the document after our editing process is complete and prior to submission to any journal. Thus, this validation process allows journal staff to distinguish changes made to the submitted manuscript after editing. If you have any questions or concerns, please contact BioScience Writers at Cert-2019@BioScienceWriters.com.

Description of Editing Service Levels offered by BioScience Writers

1. Basic
Includes simple proofreading and basic editing to achieve a grammatically correct and professionally polished scientific document. Includes correcting spelling, grammar, punctuation, verb tense consistency, noun-verb agreement, and formatting consistency.

2. Advanced
Includes all features of Basic service as well as editing for clarity and style at the sentence level. Includes eliminating jargon, smoothing language, and rewriting sentences to improve clarity.

3. Professional
Includes all features of Basic and Advanced service as well as extensive editing as needed to maximize clarity and impact. Includes stylistic editing at the document level. May include significant re-writing and reorganization of paragraphs to improve clarity and impact.

-- Edit As Needed --
With the "Edit as Needed" option our editors provide the level of service that is most appropriate for the document.

BioScience Writers provides editing, writing, and translation services to researchers and publishers around the world. Our stringent quality control and experienced native English-speaking Ph.D. and M.D. editors provide the highest quality editing services available. We do not employ students or non-scientific-degreed editors. To learn more about our premier scientific editing and copyediting services, please visit our website at www.BioScienceWriters.com.
